# Trajectories of socioeconomic inequality in early child development: a cohort analysis

**DOI:** 10.1186/s12939-022-01675-8

**Published:** 2022-06-07

**Authors:** Tanja A. J. Houweling, Joost Oude Groeniger, Pauline W. Jansen, Pol van Lier, Nil Horoz, Marieke Buil, Frank J. van Lenthe

**Affiliations:** 1 Department of Public Health, Erasmus MC, University Medical Center Rotterdam, Rotterdam, The Netherlands; 2 Department of Public Administration and Sociology, Erasmus University Rotterdam, Rotterdam, The Netherlands; 3 The Generation R Study Group, Erasmus MC, University Medical Center Rotterdam, Rotterdam, The Netherlands; 4 Department of Child and Adolescent Psychiatry/Psychology, Erasmus MC, University Medical Center Rotterdam, Rotterdam, The Netherlands; 5 Department of Psychology, Education and Child studies, Erasmus University Rotterdam, Rotterdam, The Netherlands; 6 Department of Clinical, Neuro, and Developmental Psychology, Vrije Universiteit Amsterdam, Amsterdam, The Netherlands; 7 Amsterdam Public Health Research Institute, Amsterdam, The Netherlands; 8 Research Center Urban Talent, Rotterdam University of Applied Sciences, Rotterdam, The Netherlands; 9 Department of Human Geography and Spatial Planning, Utrecht University, Utrecht, The Netherlands

**Keywords:** Socioeconomic inequality, Child development, Socioemotional development, Language development

## Abstract

**Background:**

Addressing socioeconomic inequalities in early child development (ECD) is key to reducing the intergenerational transmission of health inequalities. Yet, little is known about how socioeconomic inequalities in ECD develop over the course of childhood. Our study aimed to describe how inequalities in ECD by maternal education develop from infancy to middle childhood.

**Methods:**

We used data from Generation R, a prospective population-based cohort study in The Netherlands. Language skills were measured at ages 1, 1.5, 2, 3, and 4 years, using the Minnesota Child Development Inventory. Socioemotional (i.e. internalizing and externalizing) problems were measured at ages 1.5, 3, 5 and 9 years using the Child Behavior Checklist. We estimated inequalities in language skills and socioemotional problems across the above-mentioned ages, using linear mixed models with standardized scores at each wave. We used maternal education as indicator of socioeconomic position.

**Results:**

Children of less educated mothers had more reported internalizing (*B* = 0.72, 95%CI = 0.51;0.95) and externalizing (*B* = 0.25, 95%CI = 0.10;0.40) problems at age 1.5 years, but better (caregiver reported) language skills at 1 year (*B* = 0.50, 95%CI = 0.36;0.64) than children of high educated mothers. Inequalities in internalizing and externalizing problems decreased over time. Inequalities in language scores reversed at age 2, and by the time children were 4 years old, children of less educated mothers had substantially lower language skills than children of high educated mothers (*B* = -0.38, 95%CI = -0.61;-0.15).

**Conclusions:**

Trajectories of socioeconomic inequality in ECD differ by developmental domain: whereas inequalities in socioemotional development decreased over time, inequalities increased for language development. Children of less educated mothers are at a language disadvantage even before entering primary education, providing further evidence that early interventions are needed.

**Supplementary Information:**

The online version contains supplementary material available at 10.1186/s12939-022-01675-8.

## Background

Healthy early child development (ECD) lays a critical foundation for health and wellbeing throughout the life-course [1]. Socioemotional and cognitive development influence school success, adult mental and physical health, and adult socioeconomic position (SEP) [1–4]. The circumstances in which children grow up, including the home learning environment, the quality of stimulation and the psychosocial home environment, strongly influence child development [5]. Substantial socioeconomic inequalities in the home learning and psychosocial environment have been observed [6, 7]. These may translate into socioeconomic inequalities in ECD, which -in turn- may contribute to inequalities in lifelong socioeconomic and health outcomes [4] and to the transmission of social and health (dis) advantages across generations.

While early child health and development has received considerable interest in recent years, most attention has gone to physical child health, rather than to socioemotional and language-cognitive development [3]. Furthermore, socioeconomic inequalities in these developmental domains are often not quantified, hampering our understanding of the magnitude of the problem. Finally, very little is known about how socioeconomic inequalities in ECD develop throughout childhood, as most ECD inequality studies that have been conducted are cross-sectional in nature [8–15], with only a few exceptions using longitudinal analyses [16].

Understanding how socioeconomic inequalities develop as children age, is important, given the consequences they have for inequalities in life course trajectories in health and wellbeing [2]. To aid more effective policy making, a better understanding is needed about the ages at which socioeconomic inequalities in ECD emerge, increase and/or decrease, or persist. Inequalities in child development by maternal education are of particular interest, given the potentially important role of maternal education in the home learning and psychosocial environment and its influence on childhood cognitive development [6, 17, 18].

Our study aims to describe trajectories of inequality in cognitive and socioemotional child development by maternal education using longitudinal data from Generation R, a population-based prospective cohort study.

## Methods

### Study population

We used data from the Generation R Study, a prospective cohort in Rotterdam, the Netherlands. The Generation R Study follows children from fetal life to adolescence [19]. During the inclusion period, all pregnant women residing in Rotterdam with an expected delivery date between April 2002 and January 2006 were invited to participate. Approximately 61% (*n* = 9778) of women agreed to this. These women gave birth to 9749 live-born children, of whom 7893 children were available for postnatal follow-up. For our analyses, children were included if information was available on maternal education and if information was available on at least the baseline and one subsequent assessment of any of the developmental outcomes.

### Measures

Language development was measured using the subscale ‘language development’ of the Minnesota Child Development Inventory (MCDI) [20]. This subscale was assessed at ages 1, 1.5, 2, 3, and 4 years old. At each assessment, the main caregiver was asked to indicate (answering either ‘yes’ or ‘no’) which words or sentences their child was able to say or understand. Following the scoring method of the scale, if three items in a row were answered with ‘no’, all later items were coded to ‘no’ too [20]. The sum of the total amount of ‘yes’ responses was obtained The sum scores were converted to standardized scores for each wave, i.e. using the sample mean and sample standard deviation at each wave. Standardization was done separately for boys and girls.

Socioemotional development was measured using the Child Behavior Checklist (CBCL) [21]. This questionnaire was filled in by the main caregiver when children were 1.5, 3, 5 and 9 years old. The CBCL is classified into two broadband subscales, one for internalizing (emotional) problems and one for externalizing (behavioral) problems. At each wave, the sum of each subscale was calculated. At ages 1.5 to 5 years, the pre-school form (CBCL/1½-5), consisting of 99 problem items, was used. At 9 years, the school form (CBCL/6-18), consisting of 112 problem items, was used. The main caregiver (usually the mother) was asked to rate all problem items on a three-point scale: 0 (not true), 1 (somewhat or sometimes true), and 2 (very true or often true). The sum scores of each of the subscales were converted to standardized scores at each wave, separately for each sex.

As per our research protocol, we also explored the possibility of describing inequalities in trajectories of motor development, measured using the MCDI. Unfortunately, the scale to assess gross motor skills at age 4 years old in Generation R consisted of only two items. This caused both a ceiling effect and low variance of the scores. Also at age 18 months, there appeared to be a ceiling effect. This hampered the usability of this outcome to answer our research question. Findings for motor development are reported in the appendix (Additional file 1: Table S1).

Maternal education was assessed by questionnaire during pregnancy. If educational attainment either changed throughout the subsequent years, or was missing during pregnancy, the highest reported level at subsequent waves was used for the analyses. Following the definition of Statistics Netherlands [22], educational attainment was categorized and coded as either low (primary school; lower vocational training, intermediate general school; 3 years general secondary school), middle (> 3 years general secondary school; intermediate vocational training; 1st year of higher vocational training), or high (higher vocational training; bachelor degree; higher academic education; PhD).

### Statistical analyses

To estimate inequalities in ECD at each wave, and changes in these inequalities across waves, we used linear mixed models with maximum likelihood estimation, with a dummy for each wave, and random intercept to account for repeated observations within children [23, 24]. Exact age at each wave was added as a time-variant covariate in the analyses. We estimated and visualized the trajectories by SEP and sex using the following full factorial model:

Y = B_0_ + B_SEP_ + B_Wave_ + B_Sex_ + B_ActualAge_ + B_SEP ∗ Wave_ + B_SEP ∗ Sex_ + B_Wave ∗ Sex_ + B_SEP ∗ Wave ∗ Sex_ + U_i_

The model was estimated for each outcome separately. The model gives the difference in standardized scores of the outcome between low and high SEP at each wave, and changes in these SEP differences across waves. It also allowed us to test whether there are any sex differences in the magnitude of inequality in the outcome. Because outcomes are standardized separately for boys and girls, no conclusions can be drawn about sex differences in the outcome itself, only about sex differences in inequality in the outcome – the focus of our study.

To account for selective dropout, sample weights were used in the above analyses by estimating the inverse probability weights from a logistic model of the probability of remaining in the study sample after completing the baseline measurement [25]. Maternal education, household income (continuous variable), ethnicity (migration background yes/no), maternal age at enrollment (in years), marital status during pregnancy (categories: married, living together, no partner), mental health problems during pregnancy (yes/no), child’s sex, and score of the developmental outcome of interest at baseline, were used as predictors in this logistic regression model. Sample weights were calculated separately for each outcome because of (small) differences in sample size.

## Results

For language development, data for analyses were available for 4672 children, and for internalizing and externalizing problems for 4795 children (Table 1). About 10% of children had a mother with low educational attainment, 27% had a mother with middle level educational attainment, and 64% had a mother with a high educational attainment.

**Table 1:** Description of the study sample for each developmental domain

	Language skills^﻿a^ n (% of total)	Internalizing and externalizing problems^b^ n (% of total)
N	4672	4795
high SEP	2969 (63.5%)	3070 (64%)
middle SEP	1243 (26.6%)	1275 (26.6%)
low SEP	460 (9.8%)	450 (9.4%)
Girls	2359 (50.5%)	2422 (50.5%)

^a^Assessed with MCDI

^b^Assessed with CBCL

### Language skills

**Fig. 1:**
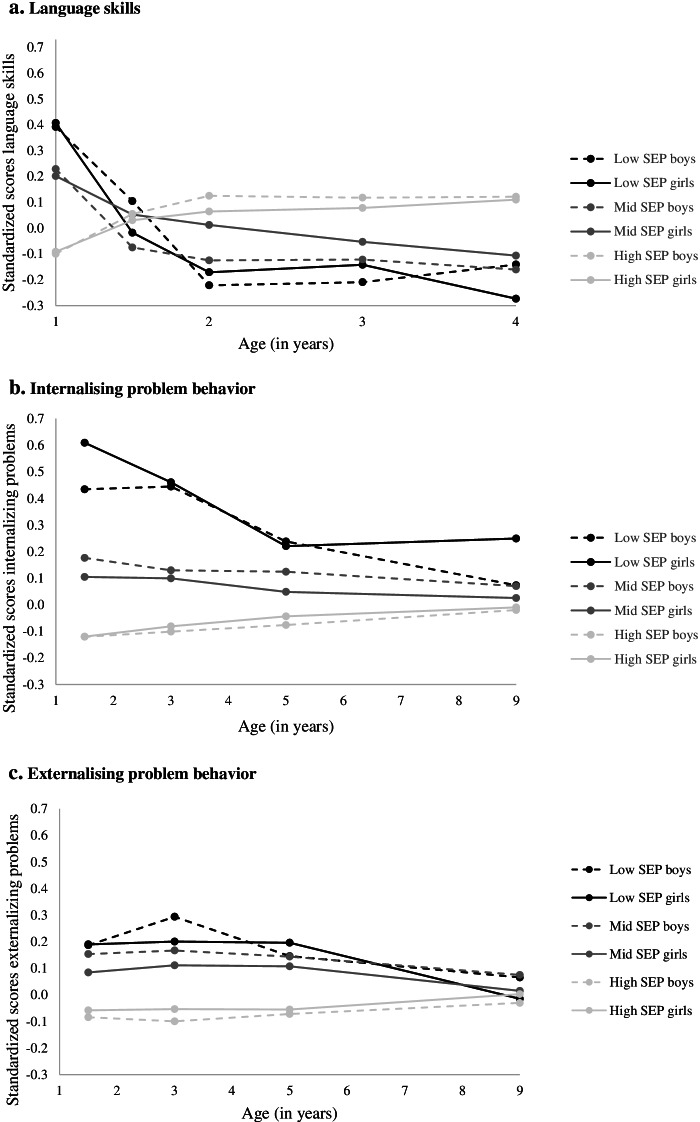
Developmental trajectories by socioeconomic position. **a** Language skills. **b** Internalizing problem behavior. **c** Externalizing problem behavior

At age 1 year old, girls of less educated mothers had a 0.50 (95%CI: 0.36;0.64) standard deviation higher score on reported language skills than girls of high educated mothers (Table 2, Fig. 1). Similar inequalities in language scores were observed for boys. Half a year later, the reported advantage of children of less educated mothers had disappeared, and from age 2 years onwards, children of less educated mothers had lower language scores than their peers of high educated mothers. At age 4 years old, girls of less educated mothers scored 0.38 standard deviation lower (95%CI: − 0.61;-0.15) on language skills than their peers of high educated mothers (middle vs. high education: -0.22, 95%CI%: − 0.32;-0.11) (*p*-value for change in inequality between ages 1 and 4 years: < 0.001). Again, findings were similar for boys (low vs. high education at age 4 years: -0.26, 95%CI: − 0.45;-0.07, p-value for change in inequality between ages 1 and 4 years: < 0.001).

**Table 2:** Socioeconomic inequalities in language skills, ages 1 – 4 years

	Age 1 year		Age 1.5 years		Age 2 years		Age 3 years		Age 4 years		Diff. between ages 1 and 4 years	
	*B* (95%CI)	*p*	*B* (95%CI)	*p*	*B* (95%CI)	*p*	*B* (95%CI)	*p*	*B* (95%CI)	*p*	*B* (95%CI)	*p*
**Language skills**												
*Girls*												
High SEP	Ref.		Ref.		Ref.		Ref.		Ref.			
Middle SEP	0.29 (0.20;0.38)	<.001	0.02 (−0.07;0.12)	.673	− 0.05 (− 0.15;0.05)	.297	− 0.13 (− 0.24;-0.03)	.015	− 0.22 (− 0.32;-0.11)	<.001	− 0.51 (− 0.64;-0.38)	<.001
Low SEP	0.50 (0.36;0.64)	<.001	− 0.05 (− 0.20;0.10)	.539	− 0.24 (− 0.42;-0.06)	.009	−0.22 (− 0.41;-0.30)	.023	− 0.38 (− 0.61;-0.15)	<.001	− 0.88 (− 1.13;0.63)	<.001
*Boys*												
High SEP	Ref.		Ref.		Ref.		Ref.		Ref.			
Middle SEP	0.33 (0.24;0.42)	<.001	−0.13 (− 0.23;-0.03)	.011	−0.25 (− 0.35;-0.15)	<.001	− 0.24 (− 0.35;-0.13)	<.001	− 0.28 (− 0.40;-0.17)	<.001	−0.61 (− 0.75;0.47)	<.001
Low SEP	0.49 (0.34;0.64)	<.001	0.05 (− 0.09;0.19)	.493	− 0.35 (− 0.51;-0.18)	<.001	− 0.33 (− 0.51;0.15)	<.001	− 0.26 (− 0.45;-0.07)	.006	− 0.75 (− 0.98;-0.53)	<.001
Difference girls-boys (*)												
High SEP	Ref.		Ref.		Ref.		Ref.		Ref.			
Middle SEP	0.04 (−0.09;0.17)	.585	− 0.15 (− 0.29;-0.01)	.033	− 0.20 (− 0.34;-0.06)	.006	−0.11 (− 0.26;0.05)	.167	−0.07 (− 0.22;0.09)	.417	−0.10 (− 0.29;0.09)	.288
Low SEP	−0.01 (− 0.21;0.20)	.944	0.10 (− 0.11;0.31)	.359	−0.11 (− 0.34;0.13)	.380	− 0.11 (− 0.37;0.15)	.421	0.12 (− 0.18;0.42)	.424	0.13 (− 0.20;0.46)	.451

*Note*:The betas give the difference in language skills between socioeconomic groups at each age. Difference are expressed in number of standard deviations. For example, a B of − 0.4 for low SEP at age 4 years means that, at this age, language skills of children of mothers with low education are 0.4 standard deviation lower than the language skills of children of mothers with a high education

(*) This gives the difference between boys and girls in the magnitude of socioeconomic inequality in the outcome, for each age

### Socioemotional development - internalizing problems

Already at age 1.5 years old, inequalities in internalizing problems were present, for both boys and girls (Table 3, Fig. 1). Girls of less educated mothers had on average a 0.72 (95%CI: 0.51;0.95) standard deviation higher score on internalizing problems than girls of high educated mothers (middle education vs. high education: 0.22, 95%CI 0.13;0.32). Boys of less educated mothers had a 0.55 (95%CI: 0.36;0.75) standard deviation higher score than boys of high educated mothers (middle education vs. high education: 0.30, 95%CI: 0.20;0.40). Inequalities in internalizing problem behavior decreased with age (*p*-value for difference between ages 1.5 and 9 years, for girls: < 0.001, for boys: 0.002). At age 9 years old, girls of less educated mothers had a 0.26 (95%CI: 0.04;0.47) standard deviation higher score on internalizing problem behavior than girls of high educated mothers, whereas inequalities between girls of middle and high educated mothers had disappeared. For boys at this age, no differences were observed between educational groups.

**Table 3:** Socioeconomic inequalities in socioemotional development from ages 1.5 to 9 years

	Age 1.5 years		Age 3 years		Age 5 years		Age 9 years		Diff. between ages 1.5 and 9 years	
	*B* (95%CI)	*p-value*	*B* (95%CI)	*p-value*	*B* (95%CI)	*p-value*	*B* (95%CI)	*p-value*	*B* (95%CI)	*p-value*
**Internalizing problem behavior**										
*Girls*										
High SEP	Ref.		Ref.		Ref.		Ref.		Ref.	
Middle SEP	0.22 (0.13;0.32)	<.001	0.18 (0.08;0.28)	<.001	0.09 (−0.01;0.19)	.065	0.04 (− 0.07;0.14)	.524	− 0.19 (− 0.31;-0.06)	.003
Low SEP	0.72 (0.51;0.95)	<.001	0.54 (0.34;0.74)	<.001	0.26 (0.09;0.44)	.003	0.26 (0.04;0.47)	.018	−0.47 (− 0.74;-0.20)	<.001
*Boys*										
High SEP	Ref.		Ref.		Ref.		Ref.		Ref.	
Middle SEP	0.30 (0.20;0.40)	<.001	0.23 (0.13;0.33)	<.001	0.20 (0.10;0.30)	<.001	0.09 (−0.02;0.20)	.394	−0.21 (− 0.34;-0.08)	.002
Low SEP	0.55 (0.36;0.75)	<.001	0.55 (0.35;0.74)	<.001	0.31 (0.13;0.50)	.001	0.09 (−0.12;0.31)	.111	−0.46 (− 0.74;-0.18)	.002
Difference girls-boys (*)										
High SEP	Ref.		Ref.		Ref.		Ref.		Ref.	
Middle SEP	0.07 (−0.07;0.21)	.306	0.05 (−0.09;0.19)	.474	0.11 (−0.03;0.25)	.134	.06 (−0.10;0.21)	.486	−0.02 (− 0.20;0.16)	.846
Low SEP	−0.17 (− 0.47;0.12)	.247	0.003 (− 0.28;0.29)	.979	0.05 (− 0.20;0.30)	.694	−0.17 (− 0.47;0.14)	.284	0.01 (− 0.38;0.40)	.964
**Externalizing problem behavior**										
*Girls*										
High SEP	Ref.		Ref.		Ref.		Ref.		Ref.	
Middle SEP	0.14 (0.05;0.24)	.003	0.16 (0.07;0.26)	.001	0.16 (0.07;0.26)	.001	0.01 (−0.09;0.11)	.807	−.13 (− 0.25;-0.16)	.026
Low SEP	0.25 (0.10;0.40)	.001	0.25 (0.07;0.43)	.006	0.25 (0.08;0.42)	.004	−0.02 (− 0.21;0.18)	.864	−.26 (− 0.48;-0.05)	.014
*Boys*										
High SEP	Ref.		Ref.							
Middle SEP	0.24 (0.14;0.33)	<.001	0.27 (0.17;0.37)	<.001	0.22 (0.12;0.32)	<.001	0.10 (0.00;0.21)	.049	−0.13 (−0.25;-0.01)	.029
Low SEP	0.27 (0.12;0.43)	.001	0.39 (0.21;0.57)	<.001	0.22 (0.07;0.37)	.005	0.10 (−0.10;0.29)	.341	−0.18 (− 0.41;0.05)	.133
Difference girls-boys (*)										
High SEP	Ref.		Ref.		Ref.		Ref.			
Middle SEP	0.09 (−0.04;0.23)	.168	0.10 (−0.04;0.24)	.147	0.05 (−0.08;0.19)	.449	0.09(−0.05;0.24)	.207	−0.002 (− 0.13;0.14)	.977
Low SEP	0.02 (−0.19;0.24)	.826	0.14 (−0.11;0.39)	.279	−0.03 (− 0.26;0.20)	.775	0.11 (− 0.16;0.39)	.426	0.09 (− 0.22;0.40)	.578

*Note*:The betas give the difference in problem behavior between socioeconomic groups at each age. Difference are expressed in number of standard deviations. For example, a B of 0.5 for low SEP at age 3 years means that, at this age, problem behavior of children of mothers with low education is 0.5 standard deviation higher than that of children of mothers with a high education

(*) This gives the difference between boys and girls in the magnitude of socioeconomic inequality in the outcome, for each age

### Socioemotional development - externalizing problems

Also for externalizing problems, inequalities were already present at age 1.5 years old (Table 3, Fig. 1), with girls of less educated mothers scoring 0.25 standard deviation higher (95%CI: 0.10;0.40) than those of high educated mothers (middle vs. high education: 0.14, 95%CI: 0.05;0.24). These inequalities were of similar magnitude for boys. Inequalities in externalizing problems remained present until 5 years of age, after which they declined (*p*-value for difference between ages 1.5 and 9 years, for girls: 0.014, for boys: 0.133). At age 9 years, inequalities were no longer present for girls. For boys, inequalities between those of less educated and high educated mothers were small (low vs. high education: 0.10, 95%CI: − 0.10;0.29), whereas there were no inequalities between the middle and high educated groups.

## Discussion

In a large, longitudinal sample with repeated assessments, we showed that inequalities in ECD differ by developmental domain: whereas inequalities in socioemotional development decreased over time, inequalities in language skills increased. More specifically, we found that children of less educated mothers had more reported internalizing and externalizing problems at 1.5 years old, but better reported language skills at 1 year old than children of high educated mothers. For internalizing and externalizing problem behavior, inequalities decreased over time. For language skills, inequalities increased over time, such that at age 4 years, children of less educated mothers had substantially worse language skills than children of high educated mothers.

### Methodological considerations

To our knowledge, our study is among the first to model inequalities in trajectories of development, across multiple developmental domains, throughout childhood using a longitudinal sample. Trajectory modeling requires repeated measurements; however, age dependent scales differ in (numbers of) items at each wave, complicating the description of the trajectories. To deal with this complexity, we used linear mixed models with standardized outcome data at each wave. This has consequences for the interpretation: the standardized scores show the changes in inequality in child development in a relative, rather than in an absolute, way. Other scoring techniques, such as mean scores of overlapping items [26, 27], percentage maximum possible (POMP) scores [28], and percentile/ranking scores [29] were considered inappropriate, because they were either not possible (e.g. for language development there were no items that overlapped between all measurement occasions), or would remove the variance in our sample. More research is needed to better understand what the most appropriate method is to model ECD trajectories with age dependent scales, as there currently seems to be no consensus on how to handle such data.

A second consideration is that the outcomes were reported by the main caregiver. We used well-known and validated measures of language and socioemotional development in young children - the CBCL and MCDI [21, 30]. In population-based studies, parental reports are most commonly used to measure language and socioemotional development in pre-school children, and were the only way to measure trajectories across the age groups in our study. Still little is known about potential educational differences in the validity of parent-reported measures of socioemotional and language development in young children. It is known that the validity of self-reports of some outcomes, such as smoking behavior, can differ between socioeconomic groups [31, 32]. To what extent this is also the case for developmental outcomes of children, merits further investigation.

A final methodological consideration is the underrepresentation of children of low SEP in our sample. In Generation R, 13% of women had a low education at enrollment in pregnancy [33]. Since we excluded children without postnatal measurements, this unbalance increased slightly: 12% of the children in our sample had a mother with low educational attainment. Although we controlled for selective attrition using inverse probability weights, these weights are not able to control for the larger problem of selective participation at enrollment. This is reflected by the fact that the unweighted results were essentially the same as the weighted ones. To the extent that our study suffers from selection bias, this has likely resulted in an underestimation of inequalities in child development, as self-selection of children with fewer problems is arguably stronger in children of lower SEP.

### Interpretation of the findings

We found that inequalities in socioemotional development decreased over time, and for externalizing problems even disappeared in later childhood. This disappearance of inequalities is not necessarily in accordance with previous research. While one longitudinal study also showed that inequalities in children’s socioemotional problems slightly decreased as children get older [34], cross-sectional studies have reported inequalities in socioemotional problems for all age groups, including later childhood (age 9 – 13 years) [35, 36]. The discrepancy between these results and our findings is possibly due to the above-mentioned selection bias in our sample, which led to an underestimation of inequalities in ECD.

The widening of inequalities in cognitive development conceivably reflect the accumulating and/or increasing effect of socioeconomic inequalities in the home learning environment [5–7]. The quality of cognitive stimulation sculpts the developing brain [37, 38]. The effects of these inequalities in the home environment are arguably compounded by lower attendance in high-quality early childhood care and education programs by children of lower socioeconomic groups [39, 40]. It remains unclear whether bilingualism may have also played a role. In the low education group, mothers with a migration background are over-represented in the Generation R cohort. However, the evidence on the role of bilingualism in language acquisition and cognitive development remains mixed and under debate [41–43].

Our finding of contrasting trends in inequality – with decreasing inequalities in socioemotional problems and increasing inequalities in language development (at least until the age of 4 years) – suggests that the influence of SEP increases or accumulates over time for cognitive outcomes such as language development, but not for socioemotional outcomes. This is in line with cross-sectional findings that SEP is more strongly associated with cognitive than with socioemotional outcomes at around primary school entry [44]. It is important to examine if this trend continues throughout primary and secondary school, or if inequalities in cognitive development narrow during primary and secondary school and/or if inequalities in socioemotional development widen again during adolescence.

### Implications and future research

Our finding that inequalities in language development emerged at around 2 years of age, and increased over time, implies that children of less educated mothers are already at a cognitive disadvantage in terms of language skills when entering primary school. This is in accordance with previous research [16, 44], and it means that, at least for language development, early – pre-school – intervention is important. Research shows that children who are already at a cognitive disadvantage in early childhood, usually have poorer school success in later childhood, especially when parents are less educated, thereby contributing to the intergenerational transmission of inequality [45, 46]. It is important to examine if inequalities in language development continue to widen throughout primary and secondary school. One could hypothesize that inequalities decrease when children enter primary school because it provides a stimulating learning environment, as demonstrated by the effectiveness of early childhood education programs [46]. The opposite is, however, also possible: inequalities may stabilize or increase, due to socioeconomic differences in school quality and peer environment [47]. In general, the findings discussed above – in combination with existing evidence – suggest the need for programs with strong emphasis on early child development to ensure all children can develop to their full potential.

## Conclusions

Trajectories of inequality in ECD differ by developmental domain: whereas inequalities in socioemotional development decreased over time, inequalities in language development increased. This means that children of mothers with low educational attainment are already at a cognitive disadvantage before entering primary education, providing further evidence that interventions are needed before the age of 4 years.

## Supplementary Information

**Additional file 1: Table S1.** Socioeconomic inequalities in motor development (ages 0.5 – 4 years).

## Data Availability

Data described in the manuscript can be made available upon request to datamanagementgenr@erasmusmc.nl; requests will be discussed in the Management Team of the Generation R Study.

## Abbreviations

ECD: Early child development
SEP: Socioeconomic position
CBCL: Child Behavior Checklist
MCDI: Minnesota Child Development Inventory
POMP: Percentage maximum possible
