# Supplementary material for: Trajectories of socioeconomic inequality in early child development: a cohort analysis
Source: Int J Equity Health. 2022 Jun 7;21:79. doi: 10.1186/s12939-022-01675-8 (PMC9172194; doi:10.1186/s12939-022-01675-8)
Supplement: Supplementary file 1 — Additional file 1: Table S1. Socioeconomic inequalities in motor development (ages 0.5 – 4 years). [file 12939_2022_1675_MOESM1_ESM.docx]

| **Annex Table 1.** Socioeconomic inequalities in motor development (ages 0.5 – 4 years) | | | | | | | | | | | | |
| --- | --- | --- | --- | --- | --- | --- | --- | --- | --- | --- | --- | --- |
|  | **Age 0.5 years** | | **Age 1 year** | | **Age 1.5 years** | | **Age 2 years** | | **Age 4 years** | | **Diff. between ages 0.5 and 4 years** | |
|  | *B*(95%CI) | *p* | *B*(95%CI) | *p* | *B*(95%CI) | *p* | *B*(95%CI) | *p* | *B*(95%CI) | *p* | *B*(95%CI) | *p* |
| **Fine motor skills** | | | | | | | | | | |  | |
| *Girls* | | | | | | | | | | |  | |
| High SEP | Ref. |  | Ref. |  | Ref. |  | Ref. |  | Ref. |  |  |  |
| Middle SEP | 0.12 (0.02;0.22) | .022 | -0.04 (-0.15;0.07) | .467 | 0.04 (-0.07;0.14) | .496 | 0.01 (-0.10;0.11) | .915 | 0.03 (-0.08;0.14) | .620 | -0.09 (-0.23;0.05) | .203 |
| Low SEP | 0.26 (0.12;0.40) | <.001 | -0.07 (-0.28;0.14) | .492 | -0.02 (-0.22;0.19) | .866 | 0.06 (-0.11;0.23) | .478 | -0.07 (-0.28;0.12) | .460 | -.34 (-0.57;-0.10) | .004 |
| *Boys* | | | | | | | | | | |  | |
| High SEP | Ref. |  | Ref. |  | Ref. |  | Ref. |  | Ref. |  |  |  |
| Middle SEP | 0.13 (0.02;0.23) | .015 | -0.03 (-0.14;0.07) | .532 | 0.11 (0.01;0.22) | .037 | 0.04 (-0.07;0.15) | .510 | 0.10 (-0.02;0.21) | .094 | -0.03 (-0.17;0.11) | .672 |
| Low SEP | 0.19 (0.06;0.33) | .005 | -0.13 (-0.33;0.07) | .193 | 0.02 (-0.15;0.19) | .844 | 0.04 (-0.13;0.21) | .644 | 0.17 (-0.02;0.36) | .077 | -0.03 (-0.25;0.19) | 0.815 |
| *Difference girls-boys* | | | | | | | | | | |  | |
| High SEP | Ref. |  | Ref. |  | Ref. |  | Ref. |  | Ref. |  |  |  |
| Middle SEP | 0.01 (-0.13;0.15) | .904 | 0.01 (-0.34;0.13) | .949 | 0.08 (-0.08;0.23) | .323 | 0.03 (-0.12;0.19) | .691 | 0.07 (-0.09;0.23) | .395 | 0.06 (-0.14;0.25) | .549 |
| Low SEP | -0.07 (-.26;0.13) | .512 | -0.06 (-0.34;0.23) | .699 | 0.03 (-0.23;0.30) | .798 | -0.02 (-0.26;0.22) | .856 | 0.24 (-0.03;0.52) | .080 | 0.31 (-0.01;0.63) | .058 |
| **Gross motor skills** | | | | | | | | | | |  | |
| *Girls* | | | | | | | | | | |  | |
| High SEP | Ref. |  | Ref. |  | Ref. |  | Ref. |  | Ref. |  | Ref |  |
| Middle SEP | 0.23 (0.13;0.33) | <.001 | 0.29 (0.19;0.40) | <.001 | -0.07 (-0.18;0.05) | .263 | 0.29 (0.19;0.39) | <.001 | 0.01 (-0.10;0.12) | .821 | -.22 (-0.37;-0.07) | .003 |
| Low SEP | 0.40 (0.25;0.55) | <.001 | 0.59 (0.44;0.74) | <.001 | -0.34 (-0.57;-0.12) | .003 | 0.36 (0.20;0.53) | <.001 | -0.00 (-0.20;0.20) | .998 | -.40 (-0.65;-0.15) | .001 |
| *Boys* | | | | | | | | | | |  | |
| High SEP | Ref. |  | Ref. |  |  |  |  |  |  |  |  |  |
| Middle SEP | 0.17 (0.07;0.27) | .001 | 0.32 (0.21;0.42) | <.001 | -0.05 (-0.17;0.06) | .369 | 0.20 (0.10;0.31) | <.001 | 0.03 (-0.08;0.14) | .604 | -0.14 (-0.29;0.00) | .056 |
| Low SEP | 0.34 (0.19;0.48) | <.001 | 0.56 (0.41;0.71) | <.001 | -0.14 (-0.34;0.06) | .173 | 0.40 (0.26;0.53) | <.001 | 0.03 (-0.17;0.22) | .798 | -0.31 (-0.54;-0.08) | .009 |
| *Difference girls - boys* | | | | | | | | | | |  | |
| High SEP | Ref. |  | Ref. |  | Ref. |  | Ref. |  | Ref. |  | Ref. |  |
| Middle SEP | -0.06 (-0.20;0.79) | .389 | 0.03 (-0.12;0.18) | .729 | 0.01 (-0.15;0.18) | .866 | -0.08 (-0.23;0.06) | .262 | 0.02 (-0.14;0.17) | .835 | 0.08 (-0.13;0.29) | .456 |
| Low SEP | -0.06 (-0.28;0.15) | .551 | -0.03 (-0.25;0.19) | .782 | 0.20 (-0.10;0.50) | .184 | 0.33 (-0.18;0.24) | .757 | 0.03 (-0.26;0.31) | .858 | .09 (-0.25;0.43) | .603 |
|  |  | |  | |  | |  | |  | |  | |

***Note****: The betas give the difference in motor skills between socioeconomic groups at each age. Difference are expressed in number of standard deviations.*

*(*) This gives the difference between boys and girls in the magnitude of socioeconomic inequality in the outcome, for each age.*
